# Hippocampal Dendritic Spines Are Segregated Depending on Their Actin Polymerization

**DOI:** 10.1155/2016/2819107

**Published:** 2016-01-10

**Authors:** Nuria Domínguez-Iturza, María Calvo, Marion Benoist, José Antonio Esteban, Miguel Morales

**Affiliations:** ^1^Institut de Neurociències, Departament de Bioquímica i Biología Molecular, Facultat de Medicina, Universitat Autonoma de Barcelona, 08193 Barcelona, Spain; ^2^VIB Center for Biology of Disease, KU Leuven, 3000 Leuven, Belgium; ^3^Center for Human Genetics and Leuven Institute for Neuroscience and Disease, KU Leuven, 3000 Leuven, Belgium; ^4^Advanced Optical Microscopy Unit, Scientific and Technological Centers, Medical School, University of Barcelona, 08036 Barcelona, Spain; ^5^INMED, Unite Mixte de Recherche 901, INSERM, Aix-Marseille Université, 13009 Marseille, France; ^6^Molecular Neurobiology Department, Centro de Biología Molecular Severo Ochoa (CSIC/UAM), 28049 Madrid, Spain

## Abstract

Dendritic spines are mushroom-shaped protrusions of the postsynaptic membrane. Spines receive the majority of glutamatergic synaptic inputs. Their morphology, dynamics, and density have been related to synaptic plasticity and learning. The main determinant of spine shape is filamentous actin. Using FRAP, we have reexamined the actin dynamics of individual spines from pyramidal hippocampal neurons, both in cultures and in hippocampal organotypic slices. Our results indicate that, in cultures, the actin mobile fraction is independently regulated at the individual spine level, and mobile fraction values do not correlate with either age or distance from the soma. The most significant factor regulating actin mobile fraction was the presence of astrocytes in the culture substrate. Spines from neurons growing in the virtual absence of astrocytes have a more stable actin cytoskeleton, while spines from neurons growing in close contact with astrocytes show a more dynamic cytoskeleton. According to their recovery time, spines were distributed into two populations with slower and faster recovery times, while spines from slice cultures were grouped into one population. Finally, employing fast lineal acquisition protocols, we confirmed the existence of loci with high polymerization rates within the spine.

## 1. **Introduction**

Dendritic spines are specializations of glutamatergic synapses. They have been the object of theoretical and experimental studies for more than a century. First identified by Ramón y Cajal [1], their role in synaptic transmission is still under study. Morphologically, spines are clearly identified as tiny protrusions, about one micron long, with a mushroom-like shape, although this static description does not reflect their great variability in size and shape. Spines are dynamic structures that undergo morphological changes in a developmental and activity-dependent manner [2, 3]. In this sense, neurons are able to control synaptic efficiency by adjusting the size and density of spines [4], which have been accepted, in turn, as important regulators of synaptic plasticity, learning, and memory formation [5–7]. In addition, abnormal spine shape and density have been associated with different pathologies, such as Alzheimer's disease, epilepsy, Down's syndrome, and fragile X syndrome [8, 9].

A motile actin cytoskeleton provides the required molecular substrate for the dynamic nature of spines [10]. At the ultrastructural level, dendritic spines are enriched with a branched actin network [11–13]. The actin state depends on the coordinated action of several actin binding proteins [8, 14]. Said equilibrium, that is, the proportion between filamentous and monomeric actin, can be quantified employing FRAP (Fluorescence Recovery After Photobleaching) [15], FRET (Fluorescence Resonance Energy Transfer) [16], or photoactivated actin [17].

Ongoing actin polymerization exerts a direct control over membrane receptor composition and the stability of the postsynaptic density, and it has been suggested that actin might serve as an anchor place in the synapse [18, 19]. Supporting this notion, spines contain discrete locus of polymerization often associated with postsynaptic density and receptor trafficking [17].

Besides neurons, glial cells also play an important role in synapse physiology and development. During synaptogenesis, glial cells release cholesterol and thrombospondins to increase synapse number and functionality [20]. Astrocytes not only regulate the synaptic microenvironment by removing or releasing neurotransmitters into the extracellular space [21]; they can also directly modulate synaptic transmission, synaptic plasticity [22, 23], and neurodegeneration [24]. Astrocyte interaction with synaptic spines requires physical contact, as demonstrated in electron microscopy reconstructions where 57% of the spines in a mature hippocampus are associated with astrocytes [25]. In organotypic hippocampal cultures, astrocytes rapidly extend and retract fine processes that associate and release from dendritic spines. Changes in astrocytic processes are coordinated with the stabilization of larger spines [26]. Furthermore, astrocyte protrusions are essential in the maturation and stabilization of newly forming spines, and thus astrocyte contact enhances both lifetime and morphological maturation of spines [27].

Here, we employed FRAP techniques to study actin spine dynamics in dissociated cultures of rat hippocampal neurons and organotypic slices. Our results reveal an unexpectedly high degree of variability regarding actin dynamics in individual spines. Moreover, the spine population was segregated into two groups, according to their recovery velocity rate. Additionally, we show that the presence of astrocytes in the culture can regulate actin cytoskeleton dynamics, in that, spines growing in the presence of astrocytes present a higher actin dynamics than those growing in the absence of astrocytes. Finally, we described a simple protocol to demonstrate the presence of polymerization hot spots within the spine structure.

## 2. Material and Methods

### 2.1. Hippocampal Neuronal Culture and Transfection

Primary cultures of hippocampal neurons were dissociated from postnatal (P0-P1) rat pups as previously described [28]. In brief, hippocampal neurons were grown in culture media consisting of Neurobasal medium supplemented with 0.5 mM glutamine, 50 mg/mL penicillin, 50 units/mL streptomycin, 4% FBS, and 4% B27 supplement (all from Invitrogen). Cells were plated in Mattek chambers with a 12 mm glass coverslip center (Mattek, USA), previously coated with poly-D-lysine (50 *μ*g/mL) and laminin (4 *μ*g/mL). On days 4, 7, 14, and 21 in vitro (DIV), 500 *μ*L (from a total of 2000 *μ*L) of the culture medium was replaced with 520 *μ*L of new, fresh medium. Two types of cultures were used, depending on the density of astrocytes. Under regular conditions, after the astrocytes grew to form a monolayer (usually after four days to a week in culture), a concentration of 4 *μ*M of cytosine-D-arabinofuranoside (SIGMA) was added to prevent glial cell overgrowth (this condition was referred to as “Ast high”). In the second type of cultures, the inhibitor was added after 2 days in vitro, to obtain cultures growing in the near-absence of glial cells (we referred to this type of cultures as “Ast low” condition). All neurons studied grew in Ast high conditions, unless otherwise indicated. Prior to plating, neurons were transfected with a vector plasmid encoding for the YFP/GFP fused to the N-terminus of chicken *β*-actin gene, under the control of the platelet-derived growth factor enhancer/promoter region (PDGF; vector kindly provided by Drs. Yukiko Goda and José Airas [29]). Transfection was performed by neuronal electroporation, using the electroporation rat hippocampal neuron kit from AMAXA according to the manufacturer's instructions or with a BioRad Cell electroporator system (exponential discharge protocol with the following parameters: 220 V and 950 mF and resistance fixed to infinitum; cells and plasmids were mixed in BioRad electroporation buffer). In both protocols, 10 *μ*g of plasmidic DNA was mixed with 2–4 million cells.

### 2.2. Hippocampal Slice Preparation, Culture, and Sindbis Virus Expression

Hippocampal slices were prepared from young rats of both sexes (postnatal days 6-7) as previously described [30]. Briefly, after dissection of the hippocampi in ice-cold gassed (5% CO_2_/95% O_2_) dissection solution (in mM: 10 glucose, 4 KCl, 24 NaHCO_3_, 234 sucrose, 0.5 MgCl_2_·6H_2_O, 0.7 CaCl_2_·2H_2_O, and 0.03 phenol red at pH 7.4), 400 *μ*m transverse slices were prepared using a tissue slicer. Slices were transferred to slice culture inserts (Millipore) and cultured in culture medium (Minimum Essential Media (MEM) supplemented with 20% (v/v) horse serum, 1 mM glutamine, 1 mM CaCl_2_, 2 mM MgSO_4_, 1 mg/L insulin, 0.0012% (w/v) ascorbic acid, 30 mM HEPES, 13 mM glucose and 5.2 mM NaHCO_3_ at pH 7.25, and a final osmolarity of 320 mOsm/L). Cultures were kept at 35°C. The recombinant EGFP-actin was delivered into slices using the Sindbis virus, as previously described [31]. The plasmid pSR5-EGFP-actin was prepared as described in [32]. Recombinant protein expression was typically 12–24 h.

### 2.3. Fluorescence Recovery After Photobleaching (FRAP)

Images were taken either with a TCS-SP5 or a TCS-SL laser-scanning confocal spectral microscopes (both from Leica Microsystems Heidelberg, GmbH). The inverted microscopes were equipped with an incubation system featuring temperature and CO_2_ control. All experiments were performed at 35°C and 5% CO_2_. Live images were acquired using a 63x oil immersion objective lens (NA 1.32), with a pixel size of 58 nm × 58 nm. The confocal pinhole was set at 4.94 Airy units to minimize changes in fluorescence due to GFP/YFP-actin moving away from the focus plane.

FRAP experiments were performed using the following protocol: 10 single prebleach scans were acquired at 225–300 ms intervals, followed by 10 bleach scans at full laser power, over a circular area of 2 *μ*m in diameter. During the postbleach period, 250 scans were acquired at 225–300 ms intervals, followed by 10 images acquired at 1 s time intervals. In order to resolve the initial fast recovery, some experiments were performed using the Leica fly mode acquisition; bleaching was performed during the X fly forward scan at 100% laser power. During the backward scan, fluorescence was read out with laser intensity set to imaging values (185 ms interval). Postbleach images (30–60) were acquired at the same time interval.

To avoid significant photobleaching, the excitation intensity was attenuated to ~5 to 8% of the laser power during image acquisition. Fluorescence was quantified using the Image Processing Leica Confocal Software. Background fluorescence was measured in a random field outside of the dendrite and subtracted from all the measurements. Dendrite fluorescence was determined for each image and compared with the initial dendrite fluorescence to determine the spontaneous signal lost during imaging.

The fluorescence signal measured in the region of interest (ROI) and normalized to the change in dendrite fluorescence was determined to be *I*_rel_ = *I*_*t*_/*I*_0_ *∗T*_0_/*T*_*t*_, where *I*_*t*_ is the average intensity in the region of interest at time *t*; *I*_0_ is the average intensity in the region of interest during prebleach, *T*_0_ is the dendrite intensity during prebleach, and *T*_*t*_ is the dendrite intensity at time *t*. The introduction of the correction factor (*T*_0_/*T*_*t*_) accounts for possible small fluctuations in total fluorescence intensity caused by the bleach itself and yields a more accurate estimate of the fluorescence measured in the ROI.

The net fluorescence recovery (mobile fraction, MF) measured in the region of interest was determined as MF = (*F*_end_ − *F*_post_)/(*F*_pre_ − *F*_post_), where *F*_end_ is the ROI mean intensity at the steady-state, *F*_post_ represents ROI intensity after photobleaching, and *F*_pre_ is the mean ROI intensity prebleach.

Each individual spine recovery curve was fitted by a two-component exponential equation, although the initial fast component, driven by diffusion, was negligible in most of the recordings. Therefore, the recovery time constant (tau, *τ*) was calculated from the fitting to a monoexponential curve.

Ultrafast recordings were performed employing a x, t acquisition mode. This protocol permits linear scans of 200–300 nm width at 1-ms intervals. For these experiments, three consecutive scans or jobs were acquired (each consisted of 2000 lines × 512 pixels in width): an initial prebleach job (2000 lines), a bleach protocol (2000 lines at maximal laser power), and a final 6 x jobs (2000 lines each), to account for a recovery time of 12 seconds. To avoid significant photobleaching, excitation intensity was attenuated to ~5 to 8% of the laser power during image acquisition.

Latrunculin A, Cytochalasin D, and Jasplakinolide were from SIGMA.

### 2.4. Two-Photon Fluorescence Imaging of Hippocampal Slice Preparations

Organotypic hippocampal slices (3–7 DIV) expressing EGFP-actin were perfused with ACSF at 30°C. Two-photon fluorescence images were obtained with a Zeiss LSM510 laser-scanning microscope using a 63x water immersion objective and a Mai Tai DeepSee (Spectra Physics) 910 nm laser as light source for excitation. Digital images were acquired using Zen software. For FRAP experiments, images were acquired every 200 ms for 2.7 min (810 images). After 3 images, the EGFP-actin signal from dendritic spines was photobleached with one iteration of high laser intensity. Fluorescence values at the spine were normalized to those of the adjacent dendrite to compensate for ongoing bleaching during imaging. Fluorescence values and the spine area were analyzed using Image J.

### 2.5. Immunocytochemistry

Immunocytochemical analysis was performed as follows: cultures were rinsed in phosphate buffer saline (PBS) and fixed for 15 min in 4% paraformaldehyde in PBS. Coverslips were then washed three times in PBS and incubated for 30 min in blocking solution (2% goat serum, 2% serum albumin, and 0.2% Triton X-100 in PBS). Antibodies were diluted in blocking solution and incubated for 60 min. GFAP and Synapsin antibodies were from Abcam (rabbit polyclonal reference 7260) and Cell Signaling USA (rabbit polyclonal reference 2312), respectively. Samples were subsequently washed three times in PBS and incubated for 30 min in PBS solution containing the appropriate fluorescence-conjugated secondary antibodies (all from Molecular Probes) and were then washed five more times with PBS buffer and mounted using Mowiol. 

### 2.6. Statistical Analysis

Statistical analyses were performed using GraphPad Prism software (GraphPad Software Inc., San Diego, CA, USA). A two-way ANOVA with Tukey's multiple comparison test was performed to detect differences in mobile fraction between neurons, cultures, and distance from somas. A one-way ANOVA was employed to study differences among neurons or between individual neurons and the whole population. A Kolmogorov-Smirnov test was performed to compare cumulative frequency distributions for spine head areas between “Ast high” and “Ast low” conditions. A Mann-Whitney test was used to test for differences between MF in both culture conditions (“Ast high” and “Ast low”) or between the culture (“Ast high”) and slices. The significance level was set at *p* < 0.05.

A better model of tau distribution was determined by comparing a single Gaussian model versus a sum of two, employing an extra sum-of-squares *F* test in GraphPad Prism. 

For all our experiments in “Ast high” conditions, a minimum of 10 neurons from around 5 independent cultures and approximately 212 spines were analyzed. For the “Ast low” condition, we studied a minimum of 30 neurons from 10 independent cultures with more than 100 spines analyzed. 

## 3. Results

### 3.1. Hippocampal Dendritic Spines Are Enriched in Actin

Culture hippocampal neurons produce dendritic protrusions with distinct stages of morphological progression [33, 34]. Dendritic filopodia could be observed as early as 6 DIV and became abundant around 9 DIV. By DIV 14, the dominant dendritic protrusions were thin spines, characterized by a relatively long neck and a small head. Mature, mushroom-shaped spines became abundant at about 18–21 DIV.

**Figure 1:**
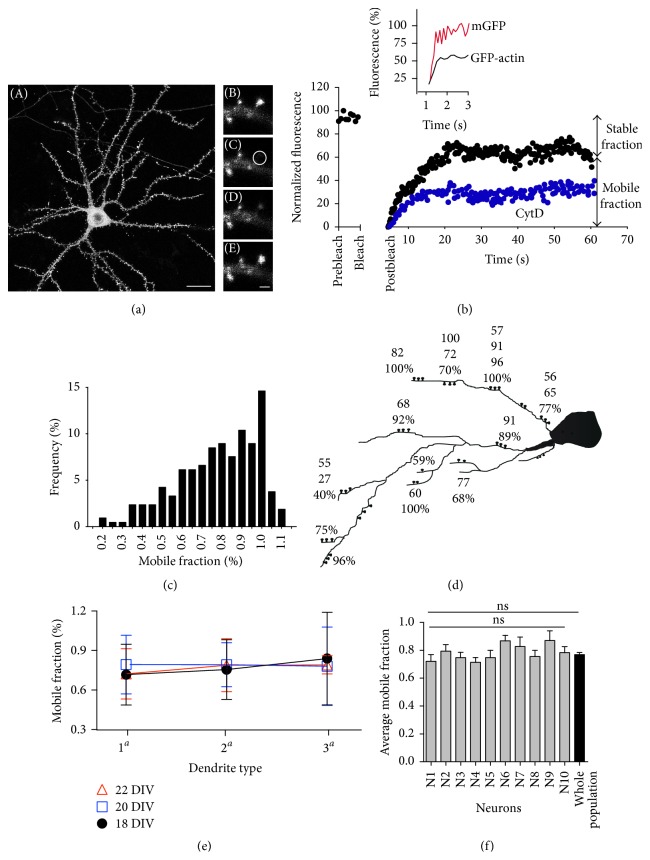
Mobile fraction values do not correlate with culture age or dendrite localization. (a) (A) An example of hippocampal neurons (growing in the presence of astrocytes) transfected with GFP-actin. Scale bar: 20 *μ*m. Pictures on the right (B to E) are higher magnification images showing prebleach (B), bleach (C), postbleach (D), and late phase of recovery (E) (60 s). Scale bar: 1 *μ*m. (b) Normalized fluorescence recovery curve of (a), showing the two fractions of fluorescence recovery: stable and mobile fractions (*n* = 212 spines, black circles). An example of the recovery in the presence of Cytochalasin D (CytD) (5 *μ*M) in the extracellular solution (*n* = 4 spines, blue circles). Top insert: comparison of the initial phase of GFP-actin (black line) and monomeric GFP (red line) recovery curves. Note the similarities between both initial phases in the first 1–3 ms. (c) Graph frequency distribution of mobile fractions from neurons 20 DIV growing in the presence of astrocytes. Mean average was 0.78 ± 0.01 (*n* = 10 neurons, *n* = 5 independent cultures, and *n* = 212 spines). (d) Neuronal structure drawing indicating the localization of the recorded spines. Note the variability in MF values along neuronal dendrites. (e) Mobile fraction values were averaged according to their dendrite type (primary, secondary, or tertiary), and the same value was calculated at days 18, 20, and 22 in vitro (*n* = 10 neurons, *n* = 5 independent cultures, mean ± SEM). As the graph shows, no differences were found for culture age or dendrite localization (two-way ANOVA with Tukey's multiple comparison test, ns). (f) Average mean of mobile fraction values from ten individual neurons was compared with the overall population of MF mean. No statistical differences were found among neurons or when each neuron was independently compared to the whole population. Only values from 20 DIV were used in this analysis (one-way ANOVA, ns) (*n* = 10 neurons, *n* = 5 independent cultures, mean ± SEM).

Several studies have reported that transfected neurons accumulate GFP-actin at dendritic spines, making them clearly visible without affecting synaptic transmission [15, 35, 36]. We transfected cultured hippocampal neurons with a plasmid encoding GFP-actin under the control of a neuronal PDGF (platelet-derived growth factor) promoter to avoid overproduction and toxicity of GFP-actin (Figure 1(a)) [28, 29]. In these neurons, growing for more than 18 DIV, staining with rhodamine phalloidin largely colocalizes with GFP-actin positive dendritic spines (90% of colocalization, data not shown). This result is consistent with the reported enrichment of actin filaments in the spines [37, 38].

To characterize actin dynamics at the spine, we have employed FRAP as previously described [39, 40]. Our approximation is based on the work of Star et al. [15] and Koskinen et al. [36]. Basically, we are assuming that (1) actin monomers are free to move in and out of the spine compartment and (2) most of the actins in the spine are in filamentous form, and these are in a dynamic equilibrium, continuously poly- and depolymerizing. Therefore, the net recovery of fluorescence at the steady-state (the so-called mobile fraction, MF) includes the free diffusion of actin monomers, plus the proportion of filaments in dynamic equilibrium. Assuming that actin monomer diffusion is constant [41], a low proportion of stable filaments should render high values of mobile fraction, and, conversely, a high proportion of stable actin filaments would produce lower mobile fraction values (Figure 1(b)). Finally, the fluorescence recovery rate is proportional to the velocity of actin monomer incorporation to the plus ends of filament, making FRAP a suitable technique to measure actin treadmilling (Figure 1(b)) [36].

### 3.2. The Mobile Fraction Value Is Specific to Each Individual Spine

In agreement with the previously reported studies, the recovery curve has two clearly distinguished components, each adjusted to a single exponential curve. The fast (initial) component showed a mean time constant of 0.61 ± 0.09 s. Similar time constant diffusion was obtained when spines from monomeric GFP-transfected (mGFP) neurons were analyzed (0.53 ± 0.093 s), supporting the idea that this fast component was driven by pure diffusion (Figure 1(b) insert and Figure 3(e)). The first component was only uncovered when a fly mode acquisition was employed and was ignored in most of the experiments because it does not provide any information about actin cytoskeleton dynamics. The second component was mostly driven by actin polymerization; consistently, Cytochalasin D (5 *μ*M) treatment, a barbed-end capping drug, reduced the mobile fraction to 0.30 ± 0.13 and slowed recovery fluorescence time as previously described (Figure 1(b); [15]). Jasplakinolide 1 *μ*M treatment, a membrane permeable actin filament stabilizer, greatly impairs fluorescence recovery (MF: around 5%; statistically nonsignificant), further confirming that the slower component depends of F-actin polymerization.

The population of mobile fraction values follows a continuous distribution (values range from 0.2 to 1.1; recovery values higher than 110% were discarded), with a mean value of 0.78 ± 0.01 (Figure 1(c)). When several spines from the same neuron were analyzed, we observed a large variability of MF values within a single neuron (see Figure 1(d) as an example). Therefore, our first question concerned the origin of this variability. Was the mobile fraction regulated by the age of the culture or by the proximity of the spine to neuronal soma? Thus, spine mobile fractions were analyzed for a period of five days (18, 20, and 22 days in culture) and MF values were averaged and segregated, according to their dendritic origin (primary, secondary, and tertiary dendrite) and age of the culture. The results (Figure 1(e)) indicate that neither age of the culture nor the distance from the cell body affects spine mobile fraction (similar mean values were obtained when spines were segregated in 20 *μ*m intervals, data not shown). Despite variability, no differences were found when average mobile fraction values were compared among neurons or between single neurons and the whole population of MFs (Figure 1(f)). In summary, considering these results as a whole, we assume that mobile fraction variability can be attributed to the individual spines themselves, and not to neurons or culture age.

### 3.3. Astrocyte Contacts Modulate Spine Actin Dynamics

**Figure 2:**
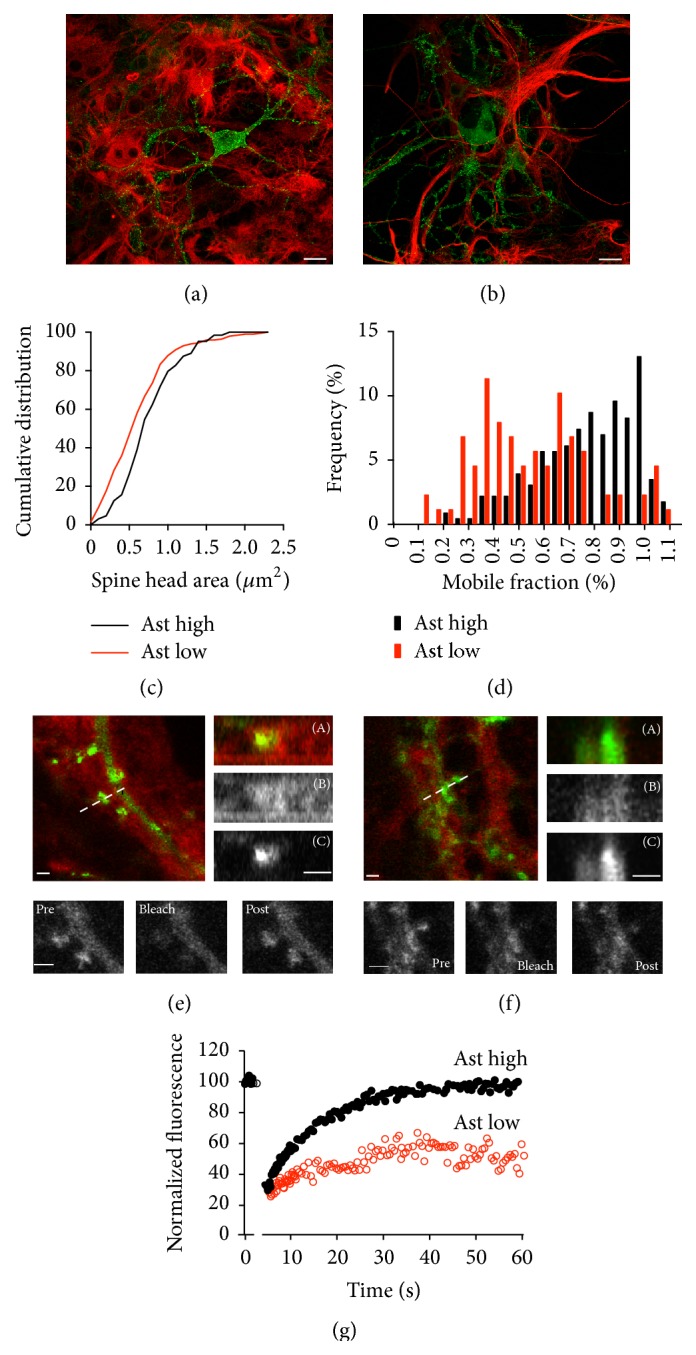
Astrocyte contact regulates actin mobile fraction. (a-b) The pictures show two neurons growing over a layer of astrocytes ((a) “Ast high” condition), or in the near-absence/absence of astrocytes ((b) “Ast low” condition). Notice how in the “Ast low” condition a large part of the neuron has no contact with the surrounding astrocytes. Neurons were transfected with GFP-actin (green) and astrocytes were identified by GFAP staining (red). Scale bar: 10 *μ*m, 21 days in vitro. (c) Cumulative distribution of spine head areas comparing “Ast high” (black) versus “Ast low” (red) (*n* = 198 spines “Ast high” and *n* = 64 spines “Ast low”) (Kolmogorov-Smirnov test, ns). (d) Frequency distribution of mobile fractions in “Ast low” conditions (red bars) (*n* = 82 spines). Of interest is that the MF was drastically reduced in the “Ast low” condition, as compared to the “Ast high” condition (Mann-Whitney test, *p* < 0.0001). The mobile fraction values distribution of “Ast high” conditions was included for comparison (black bars). (e and f) In vivo confocal images. (e) To evaluate the presence of direct astrocyte contact with the recorded spine, membranes were stained with the lipophilic dye FM4-64 (4 *μ*M, red). The dendrite shown in (e) “Ast high” condition was lying on top of an astrocytic layer. Longitudinal sections at higher magnification were performed to study spine surroundings (approximately marked as a dashed line), pictures (A) to (C) (overlap, FM4-64 and GFP-actin, resp.). Scale bar: 1 *μ*m. Bottom: detailed pictures of selected frames obtained from the FRAP experiment, prebleach, bleach, and postbleach. Scale bar: 1 *μ*m (*n* = 8). (f) Detailed picture of a dendrite growing in “Ast low” conditions. Scale bar: 1 *μ*m. Notice how FM4-64 staining was only present along the dendrite, but not surrounding the spine. A section of the optical longitudinal acquisition is shown in pictures (A), (B) and (C). Bottom: selected frames obtained from the FRAP experiment, prebleach, bleach, and postbleach. Scale bar: 1 *μ*m (*n* = 5). (g) Normalized fluorescence recovery curve of the depicted spines growing in “Ast high” (black dots) and “Ast low” (red dots). Notice that spines growing in “Ast high” conditions are characterized by a recovery close to 80%, while spines growing in “Ast low” conditions present a recovery close to 50%.

From a spurious observation, we began to suspect that the presence of astrocytes might modulate mobile fraction. Therefore, to evaluate the role of astrocytes modulating actin dynamics at the spine level, we performed FRAP experiments with two types of cultures: regular cultures growing over an astrocyte monolayer (condition: “Ast high”) and in the partial/total absence of astrocytes (condition: “Ast low”) (Figures 2(a) and 2(b)). Both types of cultures developed spines after 16 days in vitro, and recordings were made between 18 and 22 days in vitro. Despite the fact that neurons exhibit a normal growth in the absence of astrocytes, we observed a consistent reduction in basal fluorescence levels at the spines. To test whether differences arise from spine size, we analyzed the spine head area in both experimental conditions. No significant differences were found in average head area between the two conditions (“Ast high”: 0.73 ± 0.03; “Ast low”: 0.78 ± 0.04 *μ*m^2^), although the cumulative frequency distribution indicates that small spine head areas were more abundant in “Ast low” conditions (Figure 2(c)). Moreover, the initial fast component of recovery in “Ast low” conditions had a mean time value of 0.65 ± 0.04 s, similar to that obtained in “Ast high” conditions, implying that the diffusion rate is unaffected by the presence/absence of astrocytes in the culture.

In contrast, different results were observed when the mobile fraction was quantified. In “Ast low” conditions, the MF was drastically reduced (Figures 2(d) and 2(g), red open circles). Although some variability is present, the frequency distribution clearly indicates that neurons growing in these conditions have lower mobile fractions, with a range between 0.1 and 0.8 and a mean value of 0.54 ± 0.02 (Figure 2(d)).

To confirm the presence of astrocytes nearby or in close proximity to the spine, in a small number of experiments, FM4-64 (a lipophilic dye) was included in the culture media during the recording conditions. In these conditions, and without stimulated endocytosis, FM4-64 adheres to all extracellular membranes, allowing easy identification of the presence of membranes around the spine. As indicated in Figure 2(e), in “Ast high” conditions, dendrites lie on top of a membrane surface. FM4-64 staining showed a sandwich-like distribution, enfolding dendritic spines (see linear scanning in insert, Figure 2(e)(A, B, and C)), indicative of a membrane around the spine. This spine showed a mobile fraction value close to 80% (Figure 2(g), black closed circles). In clear contrast, the spine from the “Ast low” conditions was almost devoid of red fluorescence around the spine (see insert in Figure 2(f)(A, B and C)). In this case, fluorescence recovery was close to 30% (Figure 2(g), red open circles).

In addition to the physical contact between the dendritic spines and astrocytes, it is possible that glial cells release soluble factors into the medium that affect actin dynamics [42]. In order to test possible contributions of soluble factors, we studied some “Ast low” neurons treated with astrocyte-conditioned medium, but no significant effects were seen in the actin mobile fraction. Due to this absence of significant effects and the fact that the medium composition may depend on many, highly variable factors (age of astrocytes, frequency of medium replacement, degradation, etc.), we chose not to pursue these experiments any further. Nevertheless, we cannot completely rule out that some undetermined soluble factors could affect actin dynamics.

### 3.4. Spines Can Be Divided into Two Populations, according to Their Recovery Constant

**Figure 3:**
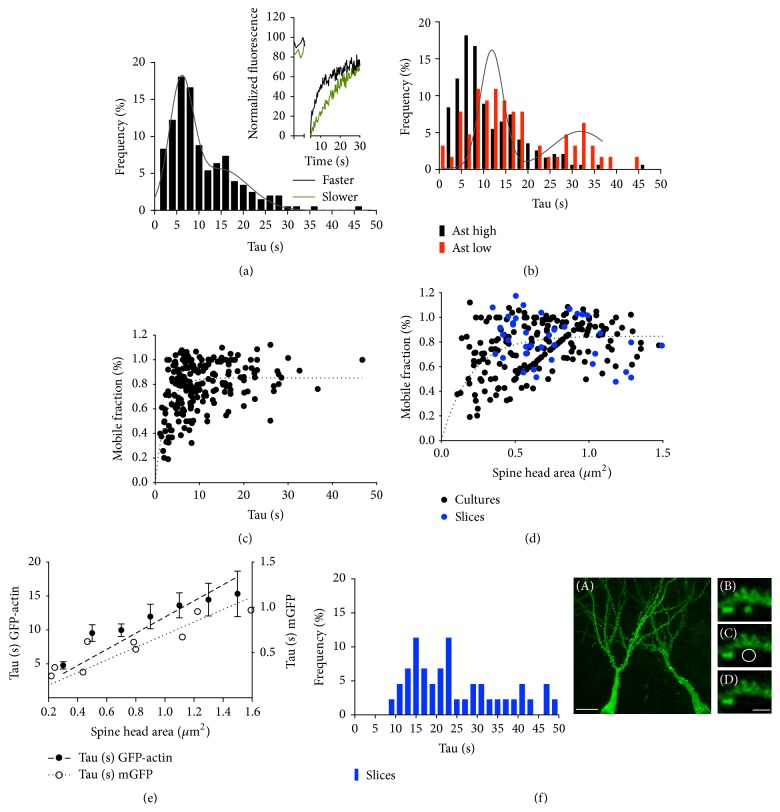
Spines can be divided into two categories, according to their fluorescence recovery time. (a) Frequency distribution of tau values in “Ast high” condition (black bars) (*n* = 205 spines). Distribution was adjusted to a two-Gaussian distribution, with two peak values of 6.02 ± 2.70 and 14.87 ± 6.32 seconds (*F* test, *p* < 0.0001) (gray line). Insert depicts two representative recovery curves for each category. Only the initial 30 seconds are displayed. Curves were normalized and scaled to the same initial time. (b) Frequency distribution of tau values in the “Ast low” condition (red bars) (*n* = 72 spines). Distribution was adjusted to a two-Gaussian distribution, with two estimates peak values of 15.12 ± 2.3 and 35.42 ± 4.22 seconds (*F* test, *p* < 0.0001) (gray line). Black bars show the frequency distribution of tau values in “Ast high” condition, plotted for comparison. (c) The mobile fraction of each “Ast high” spine was plotted against its recovery time value (dotted line, one phase exponential association). Note that spines with higher mobile fractions present slow recovery times, and vice versa. (d) Mobile fraction values of “Ast high” spines were plotted against their spine head area (black dots) (*n* = 210 spines). Of interest, spines with a larger head area show higher mobile fractions, and vice versa. Blue dots represent spines from hippocampal culture slices (*n* = 44 spines). Spines from hippocampal slices show high mobile fractions and larger spine head areas. (e) Left axis: spine head areas were grouped (0.2 *μ*m^2^ intervals) and their mean average areas were plotted against their average recovery time (*n* = 198 spines, mean ± SEM). The value distribution was adjusted to a linear regression (slope: 11.89 ± 0.80). Right axis: same relation employing spines transfected with monomeric GFP (slope: 0.69 ± 0.054) (*n* = 9 spines). Both graphs show a lineal relation between bleach area and fluorescence recovery constant, although with a tenfold difference in scale. (f) Right picture. (A) Example of two pyramidal hippocampal neurons expressing GFP-actin from an organotypic slice culture. Scale bar: 20 *μ*m. (B to D) Sequential frames of a FRAP experiment. Scale bar: 2 *μ*m. Average mobile fraction was estimated to be 0.84 ± 0.02 (*n* = 49 spines). Left graph: frequency distribution of tau values obtained from hippocampal slices (*n* = 42 spines, blue bars). Notice how slice spines fall mostly into a single distribution. Spine MF values from cultures and slices were not statistically different (Mann-Whitney test, ns).

As mentioned earlier, the recovery rate of GFP-actin fluorescence is proportional to actin polymerization velocity. To study the variability of this parameter in our neuronal population, we analyzed the rate of recovery by fitting the second component of the recovery curve to an exponential growth described by a tau value (*τ*). Recovery time values show a high degree of variability, ranging from 1.1 to 46.8 seconds. The frequency distribution graph suggests the existence of two populations of spines, determined according to their recovery time (Figure 3(a)). The frequency distribution was fitted to a double Gaussian distribution, with two average values of 6.02 ± 2.70 and 14.87 ± 6.32 seconds, respectively. The kinetics of recovery were also affected by the lack of astrocytes. In these culture conditions, constant times presented a double Gaussian distribution, with two mean values of 13.16 ± 5.84 and 33.22 ± 3.55 (Figure 3(b), red bars).

We then proceeded to evaluate whether there was any relation between recovery times and MF values (in “Ast high” condition). As shown in Figure 3(c), the relation between MF and tau values reinforces the existence of two populations of spines: one characterized by a faster recovery (up to 10 s) and lower MF values and a second population characterized by slower recovery times and higher levels of MF (Figure 3(c)). A similar distribution can be observed when mobile fraction values are plotted versus spine head size. Two populations became apparent in this graph: one with a smaller size and lower mobile fractions and a second one with larger areas and higher mobile fractions (Figure 3(d)).

Therefore, it follows that tau and spine size area are also related, with smaller spines displaying faster recovery times and larger spines being more prone to showing slower recovery times (Figure 3(e), closed dots, left axis). It can be argued that if diffusion is the main driver, the recovery time constant and photobleached area will follow a linear regression that is simply the effect of increasing the bleached area [43]. To test this hypothesis, recovery rates were analyzed for a simple diffusion process employing EGFP transfected neurons, and recovery time values were plotted against spine head areas (Figure 3(e), open circles, right axis). As Figure 3(e) indicates, recovery times are ten times slower when employing GFP-actin, which rules out diffusion as a main driver controlling actin velocity recovery.

To confirm whether this distribution of actin recovery times was a general characteristic of the spines or a peculiarity of the hippocampal cultures, we performed a similar experiment employing hippocampal organotypic slices transfected with GFP-actin (Figure 3(f)). In this condition, the estimated mean MF was 0.84 ± 0.02, which was not statistically different from the MF obtained from the cultures. The differences between these two models emerged when recovery times were analyzed. As Figure 3(f) shows, the frequency distribution of tau values from the organotypic slices indicates the presence of a single population of spines with a mean value of 25.06 ± 1.9 seconds (blue bars). Interestingly, all spines from organotypic cultures have slower recovery times. This result was confirmed when the spine area was plotted against MF. As Figure 3(d) indicates, all values from slices are segregated into a population of spines with larger areas and higher MF values (Figure 3(d), blue circles).

### 3.5. Spines Contain Polymerization Hot Spots

**Figure 4:**
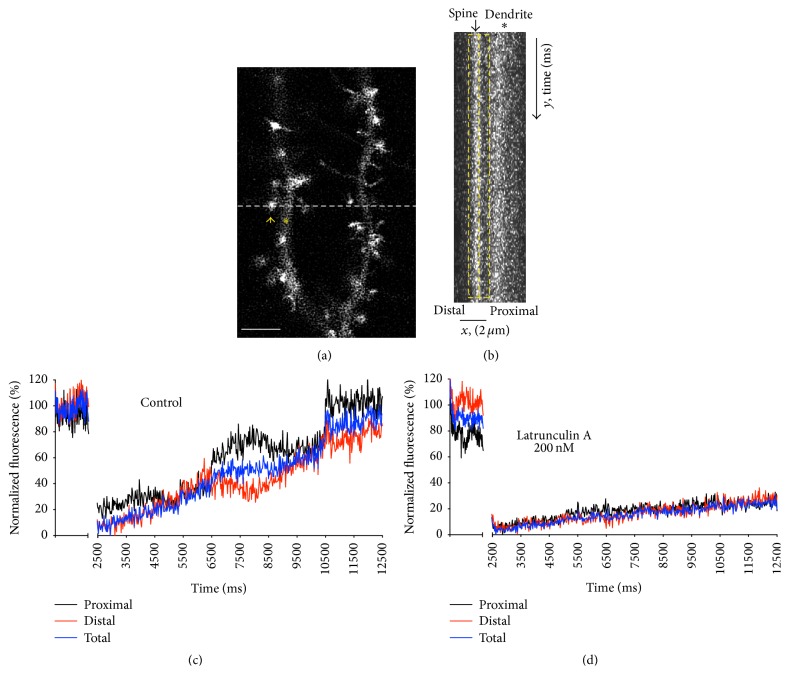
Spine head contains polymerization hot spots. (a) Example of spines from neurons in culture with astrocytes present, transfected with GFP-actin. The white line highlights the area selected for FRAP, employing a line scanning mode. In this mode, the scanned area is limited to the drawn line (around 200–300 nm wide). The spine is labeled with an arrow and the dendrite with an asterisk. Scale bar: 5 *μ*m. (b) Section of a recovery image from the initial postbleach period, obtained with the lineal acquisition mode. The *y*-axis corresponds to the acquisition time in ms (each line, 1 ms) and the *x*-axis corresponds to the localization in microns along the line. Spine and dendrite are marked with an arrow and an asterisk, respectively. Spine length was arbitrarily divided into two sections (highlighted by the yellow dashed box), corresponding to the distal and proximal part of the spine (notice how the proximal part includes the neck of the spine and a portion of the head). Scale bar: 2 *μ*m. (c) Example of normalized fluorescence recovery curve of the spine in (b). The distal part of the spine (red) and the proximal area (black) were analyzed and plotted independently. The blue line shows the fluorescence recovery curve of the entire yellow box area (sum of distal and proximal areas). In this particular example, at the times when the distal area recovery rate drops, a proportional increase at the distal area is found. Therefore, the total average fluorescence recovery does not change (blue line). Out of the 25 spines analyzed, 13 (52%) showed differences in the recovery rate between the distal and proximal areas of the spine, as shown in the example. (d) Normalized fluorescence recovery curve after addition to the culture media of Latrunculin A (200 nM), an organic compound that blocks actin polymerization by sequestering actin monomers. Notice the reduction in mobile fraction value and the absence of distributed polymerization.

Previous studies using photoactivated actin in combination with high-resolution techniques suggested the existence of polymerization hot spots along spine head structure [17]. To evaluate this point, we devised a simple experimental protocol employing conventional confocal microscopy. To this end, a line scanning mode (x, t mode) was used to perform FRAP. Employing this acquisition mode, only a narrow longitudinal area was scanned (close to 300 nm wide). This allowed us to reduce time sampling values to 1-2 ms (Figures 4(a)-4(b)). Using this acquisition mode, we were able to differentiate between the recovery rate of the distal part of the spine (cortical head area) and the proximal area (closer to the neck of the spine) (highlighted as a dashed yellow box in Figure 4(b)). When fluorescence recovery curves from each section were independently analyzed, transient changes in the slope of recovery were visible (Figure 4(c)). The changes in one area were accompanied by an equivalent alteration, but in the opposite direction, in the other areas of the spine (Figure 4(c), comparison of recovery between distal and proximal areas). A similar phenomenon was observed in 52% of the spines studied (13 of a total of 25 spines). Oscillations in the slope of recovery were observed in either the distal or proximal areas of the spine in a similar proportion, with no differences between large or small spines. Similar phenomena in the fluorescence profile were also evident during basal recording, even though the reduction of fluorescence after bleaching facilitated the discrimination (data not shown). To confirm that actin polymerization was the primary cause of these oscillations in fluorescence recovery rate, a series of experiments were performed, adding 200 nM of Latrunculin A (LatA) to the extracellular solution. LatA, an organic compound with a high affinity for monomeric actin, prevents actin polymerization by sequestering actin monomers. Despite a reduction in the recovery rate in the presence of LatA, the recovery profile was similar between the two areas of the spine head (Figure 4(d), *n* = 16). Similar results were obtained when Jasplakinolide 1 *μ*M was added to the culture media (data not shown).

## 4. Discussion

It has been proposed that a highly dynamic actin cytoskeleton in dendritic spines is necessary to support and regulate spine morphology, as well as synaptic transmission and plasticity. In the present report, we have confirmed the plastic nature of this actin cytoskeleton.

Spine actin mobile fraction values were not homogenous in either slices or cultures. On the contrary, we found a large degree of variability, with values between 20 and 100%, although the majority of spine MF values were concentrated close to 80%. Similar to the results of the pioneering work of Star and colleagues, we found that nearly all spines contained a large amount of dynamic actin [15].

Other authors have previously reported a progressive reduction in mobile fraction associated with culture aging [36]. However, our results show a large and stable MF mean value that is independent of the age of the culture or even the distance to the soma, a result that is in agreement with the lack of changes in hippocampal cultures reported by Star and colleagues [15]. Similar to this work, in our study, large spines that theoretically must bear large postsynaptic densities were associated with large mobile fractions and relatively slow actin recoveries. Confirming these findings, in hippocampal organotypic slices from 7-day-old animals, all spines had a large area and were characterized by highly mobile fraction values. However, it must be recognized that different age and culture conditions, or even FRAP protocols [44], among laboratories would certainly induce different spine actin turnovers that could contribute to explaining the discrepancies in the reported results.

The main finding of our paper is the presence of two spine populations (faster and slower recovery) in culture conditions based on their polymerization rate, and, notably, only one population in organotypic slices (slower). Spine heads typically contain a major dense network of short cross-linked and branched filaments [13]. Since Fluorescence Recovery After Photobleaching quantifies the incorporation of new fluorescent monomers, recovery time constants express, or must be proportional to, the polymerization rate. Attending to the double Gaussian distribution observed, we have classified the spines into slow polymerization (tau values between 10 and 25 seconds) and fast polymerization (between 2 and 10 seconds) groups. Interestingly, analysis of the spine head areas demonstrated that large spines were associated with slower recovery rates, while small spines displayed a faster recovery. Different molecular components at the spine ultrastructural level should easily explain the dynamic differences. A large set of actin binding proteins, such profilin II, gelsolin, debrin, and Arp2/3, have been found to be associated with the spine cytoskeleton (for a review, see Cingolani and Goda [19]). Among them, Cofilin 1/ADF has been recognized as a key regulator controlling F-actin assembly and disassembly [45]. Binding of ADF/Cofilin to actin is controlled via phosphorylation (inactivation) and dephosphorylation (activation) by LIM kinases (LIMK) and slingshot phosphatases, respectively [46], both of which are known to exert powerful control over spine morphology and synaptic plasticity [46]. Overexpression of an inactive form of Cofilin results in more mature spines through an AMPA receptor traffic-dependent mechanism [47]. Inactive Cofilin mutants increase F-actin [48] contents and reduce the actin dynamics measured by FRAP [49]. On the other hand, Cofilin 1 promotes F-actin assembly during LTP [50]; conversely, it is required for F-actin disassembly and spine shrinkage during LTD [51]. Such a dual function of Cofilin 1 thus suggests that it may be responsible, at least in part, for the observed variability among turnovers and actin mobile fractions. However, based on the complexity of the signal cascades that control actin dynamics, it is very likely that additional molecular pathways are also involved. An accurate proportion and compartmentalization of the actin binding proteins inside the spine would be crucial to ensuring proper spine morphology and function. Future experiments quantifying and analyzing the distribution of proteins controlling polymerization within the spine are necessary.

Spine size distribution was different between primary cultures and slices. In primary cultures, a large proportion of spines were smaller than their counterparts found in slices. We must keep in mind that our measurements are relative, based on an estimation of the spine area from a two-dimensional image. Levels of transfection among neurons or even the microscope employed might affect this variable. Nevertheless, a simple explanation might be the different developmental stage of spines in these two systems. Thus, young spines of small size may be more abundant and more easily found in primary cultures, while this category of spines progressively diminishes in slices until its final elimination [52]. Further experiments analyzing spine size distribution, comparing primary cultures and slices, would be needed to clarify this point.

An unexpected result was the role of astrocytes, which participated in the dynamics of the actin cytoskeleton of the spine. The absence of astrocytes shifted the actin mobile fraction distribution to smaller values and slower recoveries. A substantial series of reports have demonstrated that astrocytes play a critical role in regulating synapse formation and activity in the central nervous system [53, 54]. Astrocyte presence increases synapse formation, maturation, and stabilization [20, 27, 55, 56]. Several soluble factors secreted by astrocytes have been already identified, including thrombospondins [57], cholesterol complexes [58], and SPARC [59], which are known to be involved in synaptic formation and maturation. Moreover, the age of astrocytes in cultures regulates the probability of release and synapse maturity of cocultured neurons [60]. In addition to secreted factors, astrocytes can regulate synaptogenesis through physical interactions, and local contact by astrocytes thus elicited PKC activation by means of integrin receptor activation within the neuron, facilitating glutamatergic synaptogenesis [61]. Ephrin interactions between neurons and astrocytes have been implicated in spine morphology regulation. EphA4, a family of tyrosine kinase receptors, is enriched in dendritic spines and its ligand ephrin-A3 is localized at the astrocytic processes [56]. Acute inhibition of ephrin/EphA4 signaling in hippocampal neuronal cultures produces irregular spines with thinner heads [56]. Consistent with a role in neuron-astrocyte signaling, acute application of EphA4/Fc (which inhibits endogenous interaction of EphA4) decreases the contact lifetime between astrocyte processes and spines and reduces astrocyte-dependent stabilization of newly formed dendritic spines in organotypic hippocampal cultures [27]. Therefore, synaptic maturation and neuronal activity are among the many forms of astrocytic control. At this point, we cannot determine which signaling pathways might mediate the effect of surrounding astrocytes on the actin cytoskeleton within the spine. Nevertheless, we cannot rule out the influence of secreted factors. Further experiments will be needed to address this issue.

Finally, our experiments confirmed the existence of polymerization hot spots along the spine structure, as previously shown by Frost and colleagues [17]. In their work employing a combination of PALM techniques, the authors demonstrate the existence of discrete and separate foci along the spine head and neck, where actin polymerization velocity was elevated. The authors conclude that some of these hot spots can be associated with areas of receptor endocytosis. Our results are based on the use of fast line scans with a low spatial resolution, but a fast acquisition rate (1-2 ms). Spatial resolution is limited under these conditions. Our calculations employing fixed cells established a wide 300-nm range, limiting the measured area and therefore reducing the probabilities of detecting simultaneous hot spots. Interestingly, the presence of a polymerization hot spot was accompanied by a similar area of slower polymerization, suggesting a flux of actin monomers within the spine. This net flux of actin monomers would remain undetected when whole spine fluorescence is measured.

Synapses are inherently plastic and undergo persistent changes in strength and postsynaptic receptor composition [62]. Spine cytoarchitecture has been also associated with synaptic plasticity. Synaptic changes that support long-term plasticity (i.e., LTP) evolve through consecutive stages, and every stage involves a different set of actin functions (for a review, see Rudy [63]). Remarkably, these changes are not coupled with changes in nearby spines [64–67], supporting the functional/biochemical independence of each spine. Interestingly, the development of the two-photon glutamate uncaging technique has allowed the stimulation of a single spine while simultaneously imaging its morphology [68]. With this approach, it has been found that, upon stimulation, a single dendritic spine rapidly changes its morphology, enlarging its head for the first few minutes and eventually experiencing a whole-volume change that lasts for hours [67, 68] (for a review, see Nishiyama and Yasuda [69]).

We have observed a large degree of actin variability among spines, even on the same dendrite. This finding reinforces the notion that, at the biochemical and structural levels, each spine is self-regulated independently of its neighbors. One can speculate about the reasons for the observed variability among the spines, but an independently regulated actin cytoskeleton would indisputably subserve a large degree of systemic plasticity. In other words, every spine would independently adapt its structure to the ongoing synaptic strength, with the actin cytoskeleton being the main element responsible for these changes. As Professor Yuste proposed, the electrical and biochemical independence of each spine supports the brain's ability to form a plastic nonsaturated distributed circuit, where every spine is independently regulated [70].

It goes without saying that we are still far from having a complete understanding of actin dynamic participation in spine morphogenesis and physiology. We believe that future work must be undertaken to understand the different roles of actin binding proteins within the spine and to specifically quantify the participation of actin dynamics in the process of AMPA glutamate receptor endocytosis.

## 5. Conclusions

The main findings of our report are, first, the confirmation of the dynamic nature of the actin cytoskeleton at the spine head level. This dynamic is individually regulated by each spine, independently of neuron age or distance from the cell body. Second, we have found that the presence of astrocytes is an important regulator of the actin mobile fraction and polymerization rate. Third, according to their polymerization rate, spines can be categorized into two populations in primary cultures, or a single population in organotypic slices. Finally, our results confirm the presence of polymerization hot spots within the spine.
